# Information assessment on predicting protein-protein interactions

**DOI:** 10.1186/1471-2105-5-154

**Published:** 2004-10-18

**Authors:** Nan Lin, Baolin Wu, Ronald Jansen, Mark Gerstein, Hongyu Zhao

**Affiliations:** 1 Department of Mathematics, Washington University in St. Louis, St. Louis, MO 63130, USA; 2 Division of Biostatistics, School of Public Health, University of Minnesota, Minneapolis, MN 55455, USA; 3 Computational Biology Center, Memorial Sloan-Kettering Cancer Center, New York, NY 10021, USA; 4 Department of Molecular Biophysics and Biochemistry, Yale University, New Haven, CT 06520, USA; 5 Department of Computer Science, Yale University, New Haven, CT 06520, USA; 6 Department of Epidemiology and Public Health, Yale University School of Medicine, New Haven, CT 06520, USA; 7 Department of Genetics, Yale University School of Medicine, New Haven, CT 06520, USA

## Abstract

**Background:**

Identifying protein-protein interactions is fundamental for understanding the molecular machinery of the cell. Proteome-wide studies of protein-protein interactions are of significant value, but the high-throughput experimental technologies suffer from high rates of both false positive and false negative predictions. In addition to high-throughput experimental data, many diverse types of genomic data can help predict protein-protein interactions, such as mRNA expression, localization, essentiality, and functional annotation. Evaluations of the information contributions from different evidences help to establish more parsimonious models with comparable or better prediction accuracy, and to obtain biological insights of the relationships between protein-protein interactions and other genomic information.

**Results:**

Our assessment is based on the genomic features used in a Bayesian network approach to predict protein-protein interactions genome-wide in yeast. In the special case, when one does not have any missing information about any of the features, our analysis shows that there is a larger information contribution from the functional-classification than from expression correlations or essentiality. We also show that in this case alternative models, such as logistic regression and random forest, may be more effective than Bayesian networks for predicting interactions.

**Conclusions:**

In the restricted problem posed by the complete-information subset, we identified that the MIPS and Gene Ontology (GO) functional similarity datasets as the dominating information contributors for predicting the protein-protein interactions under the framework proposed by Jansen *et al*. Random forests based on the MIPS and GO information alone can give highly accurate classifications. In this particular subset of complete information, adding other genomic data does little for improving predictions. We also found that the data discretizations used in the Bayesian methods decreased classification performance.

## Background

Proteins transmit regulatory signals throughout the cell, catalyze large numbers of chemical reactions, and are crucial for the stability of numerous cellular structures. Interactions among proteins are key for cell functioning and identifying such interactions is crucial for deciphering the fundamental molecular mechanisms of the cell. As relevant genomic information is exponentially increasing both in quantity and complexity, *in silico *predictions of protein-protein interactions have been possible but also challenging. A number of techniques have been developed that exploit combinations of protein features in training data and can predict protein-protein interactions when applied to novel proteins. Our study is motivated by a study by Jansen *et al*. [1], who proposed a Bayesian method to use the MIPS [2] complexes catalog as gold standard positives and lists of proteins in separate subcellular compartments [3] as gold standard negatives. The various protein features considered in this method include time course mRNA expression fluctuations during the yeast cell cycle [4] and the Rosetta compendium [5], biological function data from the Gene Ontology [6] and the MIPS functional catalog, essentiality data [2], and high-throughput experimental interaction data [7-10]. The MIPS and Gene Ontology functional annotations are used for quantifying the functional similarity between two proteins. The MIPS functional catalog (or GO biological process annotation) can be thought of as a hierarchical tree of functional classes (or a directed acyclic graph (DAG) in the case of GO). Each protein is either a member or not a member of each functional class, such that each protein describes a "subtree" of the overall hierarchical tree of classes (or subgraph of the DAG in the case of GO). Given two proteins, one can compute the intersection tree of the two subtrees associated with these proteins. This intersection tree can be computed for the complete list of protein pairs (where both proteins of each pair are in the functional classification), and thus a distribution of intersection trees is obtained. Then the "functional similarity" between two proteins is defined as the frequency at which the intersection tree of the two proteins occurs in the distribution. Intuitively, the intersection tree gives the functional annotation that two proteins share. The more ubiquitous this shared functional annotation is, the larger is the functional similarity frequency; the more specific the shared functional annotation is, the smaller is the functional similarity frequency. The essentiality data represents a categorical variable that denotes whether zero, one or both proteins in a protein pair are essential. The supplementary online material of [1] provides more details about the quantification of these variables. Their Bayesian method predicts protein-protein interactions genome-wide by probabilistic integration of genomic features that are weakly associated with interactions (mRNA expression, essentiality and localization). The model was used for two separate predictions of probabilistic interactomes (PI), one of which (PIE) is built on four high-throughput experimental interaction data sets, and the other (PIP) on the mRNA expression, Gene Ontology, MIPS functional and coessentiality data. Within the PIP sub-network, different genomic features are assumed to be independent in prior. In addition, this method involved discretizing the raw data into groups and representing the two mRNA expression profiles (cell cycle and Rosetta compendium data) by their first principal component for computational convenience.

Our current study focuses on assessing the contributions of different types of genomic data towards predicting protein-protein interactions. This may help us to understand which genomic features have the closest biological relationship with protein-protein interactions and hence to construct a better prediction model. As prediction rules involving less relevant information may have lower prediction accuracy, our analysis can give us insights into how to construct more parsimonious models with comparable or better prediction accuracy. A potential disadvantage of the Bayesian network approach may be that the data discretization can obscure information contained in the raw genomic data. Thus, in addition to assessing the information content of the data sources, we also propose alternative non-Bayesian models that fully utilize the data without discretization. These methods, such as logistic regression and random forests, do not require prior knowledge, and we can evaluate the importance of the different genomic features in the context of these methods.

## Results and discussions

To accurately and quantitatively assess the information contributions of different genomic features, we construct in essence a simplified problem that has some but not all of the elements of the original study. Here, we only look at a subset of the data from [1] comprising the 18 million protein pairs in total and approximately 8,000 gold standard positives and 2.7 million gold standard negatives. This subset (see Additional File 1) contains 2,104 positives and 172,409 negatives. In this subset, we have complete information for each feature and we can thus quantitatively assess the relative contributions of the different features on this set. This data set can be downloaded from . In doing so, we find that some of the features have stronger influence on the overall prediction. While this might be true for the larger problem as well, there are a number of caveats that one has to keep in mind, such as that the features that are present in this subset might not be the strongest in the whole set of 18 million protein pairs.

### Alternative models

Here, we construct models for predicting protein-protein interactions that, given the gold standards, are basically dichotomous classifiers. Multiple logistic regression [11] is one commonly used model for such an application [12,13]. An alternative, more sophisticated supervised learning approach that we apply is the random forest algorithm [14]. Note that, although not our focus here, all these methods can be used to compute the estimated probabilities for predicted protein-protein interactions.

#### Logistic regression

The logistic model has the advantage that it provides an estimated probability that a pair of proteins interact, and is readily available in standard statistical packages. In this paper, the logistic regression analysis was generated using PROC LOGISTIC in SAS/STAT software, Version 9 of the SAS System. Moreover, we can evaluate the importance of different genomic features by variable selections. Among many available schemes, we chose stepwise variable selection that is widely used in standard packages. Stepwise selection is a greedy search algorithm that selects variables with the best marginal prediction power given the current model. To quantify the importance of the predictor variables to the model fitting, we can use the deviance measure

-2(log L_1 _- log *L*_0_),

where *L*_0 _is the likelihood of the final model given by the stepwise selection, and *L*_1 _is the likelihood of the reduced model by removing all terms that involve the corresponding predictor variable from the final model. However, this measure only considers the prediction power of variables for the training sample but not for any random test samples. Therefore, this measure can be biased due to its dependence on the training sample.

We consider, similarly as in [1], all the main effects and interaction terms among the genomic features in the PIP (indirect evidence for protein-protein interactions) and the PIE (direct experimental protein-protein interaction measurements) respectively. Table 1 presents all the terms remained in the final model and their orders to enter the final model. Table 2 shows the deviance measure of predictor variables. The Gavin data, Gene Ontology and MIPS functional similarity features, and the cell cycle gene expression data are the most important genomic evidences for predicting protein-protein interactions according to the deviance measure, whereas the three other high-throughput experimental data sets are less relevant or even do not have significant effects to be included in the final model. However, the logistic model is restricted by its linear form and may not provide an optimal solution to the prediction problem. And it will be more objective to evaluate the variable importance according to its prediction accuracy for any random test samples. In the following, we present the results from using the random forest, a more sophisticated supervised learning algorithm.

#### Random forest

The "random forest" method [14] is a supervised learning algorithm that has previously been successfully applied to many genomic studies. It has been implemented in the randomForest package of R [15]. A random forest is an ensemble of many classification trees generated from bootstrap samples of the original data. It is well known that random forests avoid overfitting and usually have better classification accuracy than classification trees. A natural way to evaluate the importance of the feature variables with the random forest algorithm is to measure the increase of the classification error when those variables are permuted. Intuitively, the more important variables will, when permuted, produce larger classification errors. The importance score provided by the random forest is a more accurate estimate of the classification error that considers the situation of random test samples. Therefore, this importance score provides a more objective evaluation of the relative merit of different genomic features on protein-protein interaction prediction. Moreover, the intrinsic tree structure of the random forest easily takes into account the interactions among the different variables and avoids complications caused by missing data that occurred in many other modeling procedures.

**Figure 1:**
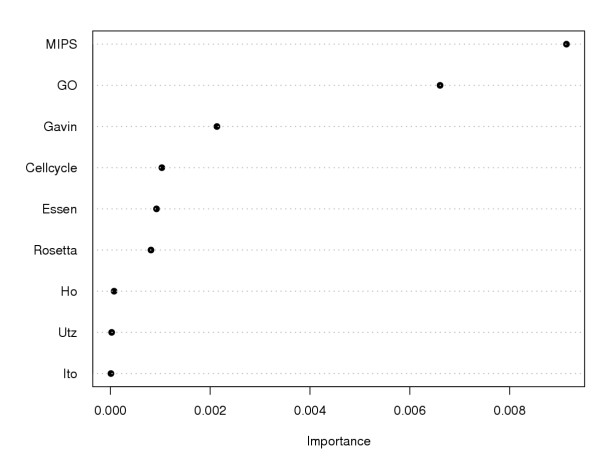
**Importance measure of genomic features from the random forest algorithm **The horizontal axis presents the importance measure whereas the vertical axis denotes the genomic features.

We performed our random forest analysis by growing 5,000 trees. Figure 1 shows the importance measures of the genomic evidences used in the random forest algorithm. The result agrees mostly with that of the logistic regression in that the MIPS and Gene Ontology functional similarity features are found to be very important, whereas most of the high-throughput experimental data sets have negligible effects. However, different from the result from logistic regression, the Gavin data set is shown to be less important than MIPS and Gene Ontology functional similarity features after considering the situation of random test samples. These observations motivated us to perform a more thorough information assessment of the genomic evidences considered. We first compared the performance of different classification methods (random forest, logistic regression and Bayesian network), and then evaluated the importance of the different genomic datasets within the framework the best method (the method with the lowest classification error).

### Comparison of three methods

**Figure 2:**
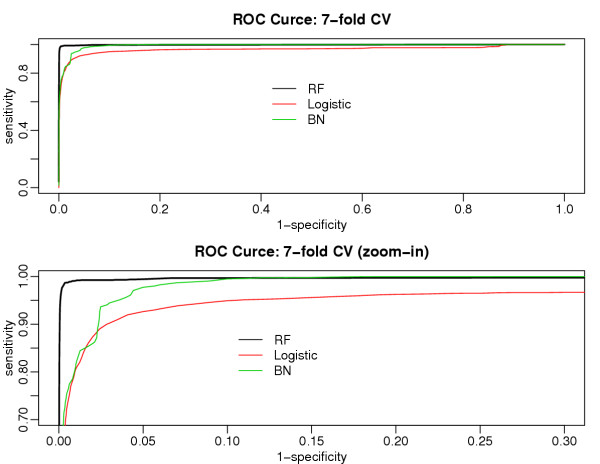
ROC curves of random forest, logistic regression and Bayesian networks using 7-fold cross validations

We conducted 7-fold cross validations on the subset with complete information (described above) on all the features for random forest, logistic regression and the Bayesian network method. Figure 2 displays their receiver operating characteristic (ROC) curves, where we observe a better performance of the random forest over the other two and similar performances between logistic regression and the Bayesian network.

### Information assessment

Information assessment of different genomic data may help us understand their relationship with protein-protein interactions, and form a guideline for future model development.

#### MIPS and gene ontology functional similarity data

**Figure 3:**
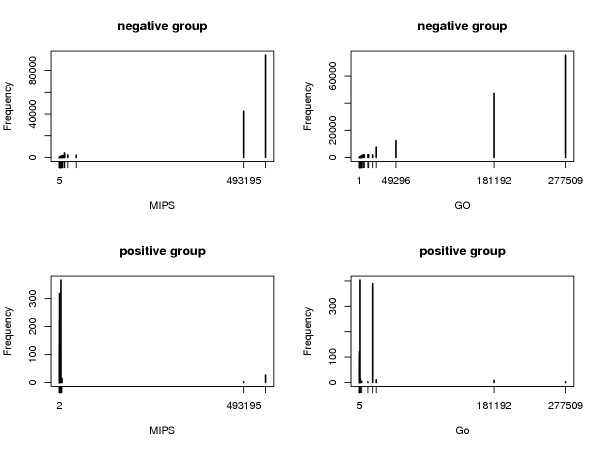
Histograms of MIPS and Gene Ontology function data for gold standard positives and negatives

**Figure 4:**
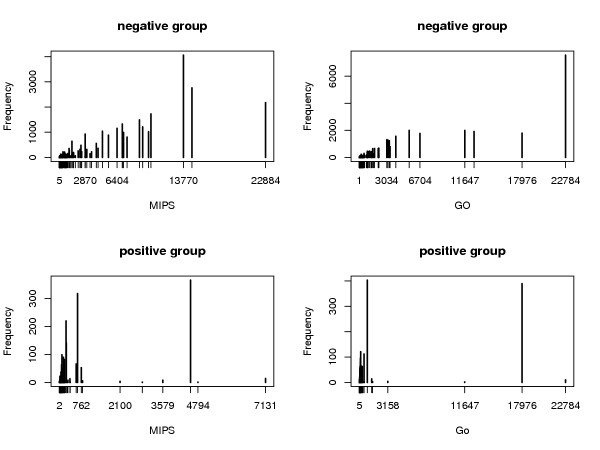
Zoom-in histograms of MIPS and Gene Ontology function data for gold standard positives and negatives on the lower end

We saw that the MIPS and Gene Ontology functional similarities were the two most important information sources under both the logistic regression and random forests methods. Histograms of the MIPS and GO functional similarity data (Figures 3 and 4) show that they are very different for the gold standard positives and negatives; protein pairs in the gold standard positives are associated with smaller functional similarity values than the gold standard negatives. This pattern explains why the functional similarity features have such a strong impact on classification accuracy in the model fitting, as observed in Figure 2. However, the vast number of protein pairs in the gold standard negatives are likely to be those that have not been thoroughly studied by researchers, and henceforth are observed to belong to large functional categories that actually should be further divided into more specific categories. This conjecture suggests that the information from MIPS and Gene Ontology function data is possibly caused by selection biases other than intrinsic biological relevance. It deserves further investigations of the relationship between the gold standards and the MIPS and Gene Ontology functional similarity data.

In the following paragraphs, we show quantitatively that the MIPS and Gene Ontology functional similarities are the dominating information contributors for predicting protein-protein interactions, while other genomic features have negligible benefit and can not provide credible predictions by themselves. We examine the performance of random forests using three different genomic feature sets: (i) all genomic features included, (ii) MIPS and Gene Ontology functional similarities only, and (iii) genomic features other than the MIPS and Gene Ontology functional similarities. The random forest performance is evaluated with the classification error (*Err*) defined as follows.

Denote *Err*_1 _as the proportion of protein pairs misclassified in the gold standard positives, and *Err*_2 _the counterpart for the gold standard negatives. Then we define the classification error as the average of *Err*_1 _and *Err*_2_.


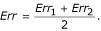


*Err *is a balanced error rate across gold standard positives and negatives. Suppose the joint probability density functions of the predictor features **X **are *f*_1_(*X*) and *f*_2_(*X*) for the gold standard positives and negatives, respectively. Denote a classifier by *C*(*X*). Then the classification error can be written as


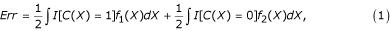


where *I*(*A*) is an indicator function equal to 1 when *A *is true and 0 otherwise. A minimal classification error *Err*_*min *_can be computed by minimizing (1) across the space of **X**. It is easy to see that


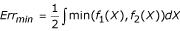


is achieved at *C*(*X*) = *I*(*f*_1_(*X*) >*f*_2_(*X*)). With this formula, we can estimate the optimal (minimum) classification error based on any estimates of *f*_1_(*X*) and *f*_2_(*X*). In our study, *f*_1_(*X*) and *f*_2_(*X*) are estimated by their empirical density functions.

Table 3 presents the optimal classification error using the MIPS and Gene Ontology functional similarity data. Using the MIPS and Gene Ontology functional similarity data sets alone results in a highly accurate classification with an optimal error of only 0.28%. Table 3 also shows the effects of the data discretizations that were originally used in the Bayesian network method ("grouped"). The significant discrepancy between optimal classification errors using the raw data and the discretized data ("grouped") suggests that the discretization causes serious loss of information.

#### Other genomic features

We also estimated the classification errors using the other genomic features within the random forest framework. Table 4 shows that adding the other genomic evidences in the complete-information subset provides only negligible benefit or even reduces the classification accuracy.

**Figure 5:**
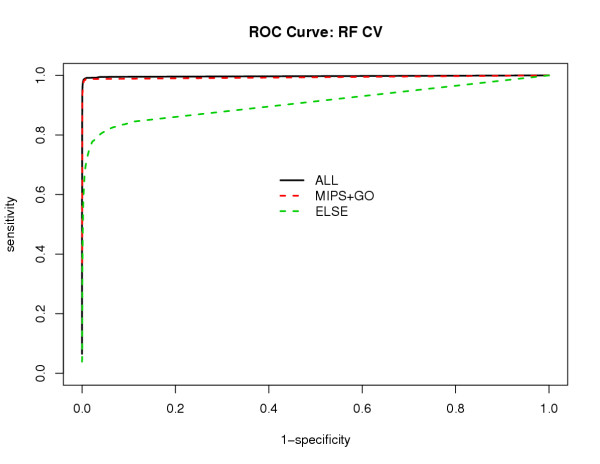
**ROC curves of random forest using different genomic feature sets **'All' – all genomic information; 'MIPS+GO' – only MIPS and Gene Ontology function data; 'ELSE' – genomic features other than MIPS and Gene Ontology function data

Moreover, we compared the ROC curves (Figure 5) of the random forest method using all genomic information, only the MIPS and GO functional similarities, and the genomic information other than MIPS and GO. Figure 5 shows that we barely gain any by considering other genomic information if the MIPS and GO are available; classifications without the MIPS and GO functional similarity data are poor on the complete-information subset. Note, however, that the subset of full interaction data which have the strongest expression correlations is not necessarily the complete-information set considered. Hence, we would expect that expression correlations might be a stronger source of information in other context.

## Conclusions

In the restricted problem posed by the complete-information subset, we identified that the MIPS and Gene Ontology functional similarity datasets as the dominating information contributors for predicting the protein-protein interactions under the framework proposed in [1]. Random forests based on the MIPS and GO information alone can give highly accurate classifications. In this particular subset of complete information, adding other genomic data does little for improving predictions. The MIPS and GO information, however, is only available for a small proportion of the ~18M protein pairs.

We considered alternative non-Bayesian methods such as logistic regression and random forest for predicting protein-protein interactions. These existing methods do not require prior information needed for the Bayesian approach, and can fully utilize the raw data without discretization. The logistic model performs similarly as the Bayesian method in terms of classifications and, like the Bayesian method, produces estimated probabilities that two proteins interact. As a dichotomous classifier, the random forest method outperforms the other methods considered and efficiently uses the information, although it is computationally more expensive. In particular, its importance measure provides a more objective assessment of different genomic features on predicting protein-protein interactions than simply considering contributions to model fitting. These findings are motivation to look for other, more sensible data resources and superior models.

We found that the data discretizations used in the Bayesian methods decreased classification performance. We note here that the genomic features datasets investigated here themselves are highly processed versions of the datasets they were derived from and that there may be better ways to take the original data into account.

Another caveat is that the predictions might be just defining groups of proteins that have the same genomic properties as the protein complexes in the MIPS data. This does not necessarily mean that they really represent protein complexes. Rather, they may represent groups of proteins that have the same properties as protein complexes.

In this analysis we have looked at the relative weights of various features in predicting protein-protein interactions based on the previous study in [1]. We looked at a particular subset of the data where we had complete information and we were able to show that, for this particular subset of the full information, we are able to show that the functional classification features in the MIPS functional catalog and Gene Ontology were the most informative and that particular machine learning algorithms, such as random forests were more effective than Bayesian networks. However, one has to keep in mind that in the full problem there is the issue of incomplete information. On data sets with incomplete information Bayesian approaches maybe more effective because they can easily handle the missing information. Further careful studies such as these will be needed to determine what the optimum machine learning method is and the optimum features are in presence of incomplete information. It will be also of great interest to consider other genomic features such as phylogenetic profiles [16] and local clustering information [17]. This is just the first step in that direction.

## Methods

### Logistic regression

Denote the gold standards by random variable Y and the other genomic features by *X*_1_, *X*_2_, ..., *X*_*n*_. Let *Y *= 1 when two proteins interact, i.e., they are in the same complex, and *Y *= 0 when not. The logistic model is of the form


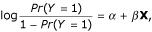


where the random vector **X **consists of *X*_1_, *X*_2_, ..., *X*_*n *_and their interaction terms.

### Stepwise variable selection

The stepwise selection procedure starts from a null model. At each step, it adds a variable with the most significant score statistics among those not in the model, then sequentially removes the variable with the least score statistic among those in the model whose score statistics are not significant. The process terminates if no further variable can be added to the model or if the variable just entered into the model is the only variable removed in the subsequent elimination. Here, the score statistic measures the significance of the effect of a variable.

### ROC curve analysis

Receiving operator characteristic (ROC) curve [18] is a graphical representation used to assess the discriminatory ability of a dichotomous classifier by showing the tradeoffs between *sensitivity *and *specificity*. *Sensitivity *is calculated by dividing the number of true positives (TP) through the number of all positives, which equals the sum of the true positives and the false negatives (FN); *specificity *is calculated by dividing the number of true negatives (TN) through the number of all negatives, which equals the sum of the true negatives and the false positives (FP).

*Sensitivity *= *TP*/(*TP *+ *FN*), *Specificity *= *TN*/(*TN *+ *FP*).

The ROC curve plot shows 1 - *specificity *on the *X *axis and *sensitivity *on the *Y *axis. A good classifier has its ROC curve climbing rapidly towards upper left hand corner of the graph. This can also be quantified by measuring the area under the curve. The closer the area is to 1.0, the better the classifier is; and the closer the area is to 0.5, the worse the classifier is.

## Authors' contributions

NL and BW conducted the major part of the data analysis, and created all the tables and figures, under the supervision of HZ. RJ and MG provided the data sets for the analysis and contributed to the discussion on the comparisons of different methods. All authors read and approved the final manuscript.

## Supplementary Material

Additional File 1 The complete-information subset in ZIP file. Click here for file

## Figures and Tables

**Table 1:** Order of variables that enter the final model by stepwise selection in logistic regression

Variables	Order
Gavin	1
MIPS	2
Rosetta	3
GO	4
cellcycle	5
essentiality	6
Rosetta*cellcycle	7
cellcycle*essentiality	8
Ho	9
GO*essentiality	10
Uetz	11
GO*cellcycle	12
GO*cellcycle*essentiality	13
MIPS*essentiality	14
MIPS*Rosetta	15

**Table 2:** Deviance of the reduced model from the final model by removing corresponding variables

Variable	Deviance
GO	1376.437
MIPS	1333.97
essentiality	579.988
Rosetta	778.493
cellcycle	1271.461
Ho	68.718
Uetz	20.513
Gavin	1839.181

**Table 3:** Optimal classification errors when using different genomic features

Variables	Optimal Classification Error
MIPS	1.69%
GO	2.15%
MIPS+GO	0.28%
MIPS (grouped)	7.31%
GO (grouped)	13.35%
MIPS+GO (grouped)	6.34%

**Table 4:** Classification errors of the random forest algorithm when using different genomic features

Variables	*Err*_1 _(positives)	*Err*_2 _(negatives)	*Err*
MIPS+GO	114/2104 = 5.42%	180/172409 = 0.1%	2.76%
ALL	165/2104 = 7.80%	89/172409 = 0.05%	3.95%
ELSE	1056/2104 = 78.09%	313/172409 = 0.20%	25.20%
